# Antecedents of picky eating behaviour in young children

**DOI:** 10.1016/j.appet.2018.07.032

**Published:** 2018-11-01

**Authors:** Pauline M. Emmett, Nicholas P. Hays, Caroline M. Taylor

**Affiliations:** a Centre for Child and Adolescent Health, Bristol Medical School, University of Bristol, UK; b Nestlé Product Technology Center - Nutrition, La Tour-de-Peilz, Switzerland

**Keywords:** Picky eating, Complementary feeding, Feeding behaviour, ALSPAC, Pre-school children, Parental feeding practices, ALSPAC, Avon Longitudinal Study of Parents and Children, FFQ, Food Frequency Questionnaire, OR, Odds Ratio, CI, Confidence Interval, FITS, Feeding Infants and Toddlers Study

## Abstract

**Background:**

Picky eating behaviour in young children is a common concern for parents.

**Objective:**

To investigate early life factors which are associated with a child becoming a picky eater.

**Design:**

Singleton children from the Avon Longitudinal Study of Parents and Children were studied prospectively (n = 5758–6608). Parental-completion questionnaires were used to define ‘picky eating’ status at age 3 years, and child and parental feeding behaviours and practices throughout the first 2 years of life. Multinomial logistic regression models with 3 levels of picky eating (*not*, *somewhat* and *very* picky) as the dependant variables tested associations with antecedent variables, from pregnancy, and the first and second year of life, separately, then combining all significant variables in a final model.

**Results:**

Feeding difficulties during complementary feeding and late introduction of lumpy foods (after 9 months) were associated with increased likelihood of the child being *very* picky. A strong predictor was the child being choosy at 15 months, particularly if the mother was worried about this behaviour. Many children (56%) were considered to be choosy at 15 months: 17% went on to be *very* picky at 3 years if the mother was not worried, compared with 50% if the mother was very worried by the choosiness. The mother providing fresh fruit and eating the same meal as the child were protective against later ‘picky eating’, while feeding ready-prepared food was predictive.

**Conclusion:**

Advice and support to parents could help to reduce picky eating behaviour. Parents should be encouraged to introduce lumpy foods by 9 months, to feed fresh foods particularly fruit, and to eat with their children. Parents should be reassured that choosiness is normal and to continue to provide a variety of foods.

## 1 Introduction

Picky eating is characterised by an unwillingness to eat familiar foods or to try new foods, as well as strong food preferences (Dovey, Staples, Gibson, & Halford, 2008). From birth infants have an innate preference for sweet and salty tastes and tend to reject sour and bitter tastes, while a savoury (umami) taste is more likely to evoke a neutral response (Mennella & Ventura, 2011). Infants are exposed to different tastes related to their mother's diet in urtero and via breast milk that may affect taste acceptance (Mennella, Jagnow, & Beauchamp, 2001). Furthermore breast milk and infant formula both have a sweet taste that is readily accepted by infants, however once complementary feeding (weaning) starts many different tastes must be accepted if the child is to learn to eat a balanced diet. Fruits and some vegetables that are sweet can be readily accepted; however, vegetables often have bitter taste notes and fruits sometimes have sour ones, and these tastes tend to be rejected at first. Infants also need to learn how to cope with different textures of food as they develop the skills required for chewing and swallowing adult foods (Gisel, 1991). Therefore, the process of introducing complementary foods can be critical in helping a child to learn to eat a well-balanced diet containing a variety of foods (Nicklaus, 2009). Parents often find this process difficult to manage and by the time children reach 3 years of age a proportion of children, ranging from 6% to 50% in various studies (C. M. Taylor, Wernimont, Northstone, & Emmett, 2015), are perceived as ‘picky eaters’ by their parents. Picky eating behaviour in children is a cause for concern for many parents and may have important consequences for nutrition and health-related outcomes in the child (Wright, Parkinson, Shipton, & Drewett, 2007; de Barse et al., 2015).

A review of the complementary feeding literature and national weaning guidelines identified three important aspects of successful complementary feeding practice that could also be key in averting the development of picky eating: the ‘when’, ‘what’ and ‘how’ (Schwartz, Scholtens, Lalanne, Weenen, & Nicklaus, 2011). The ‘when’ refers to the timing of initiation of complementary feeding and the rate and timing of introduction of different types of food. The ‘what’ includes the balance of foods and nutrients introduced and the sensory properties of the foods (taste and texture). Parent–child interactions are important for the ‘how’, as well as the child feeding themselves and regulating their own intake. Parents are acting as providers, models and controllers of child food intake during this process. Data from the UK Avon Longitudinal Study of Parents and Children (ALSPAC), a prospective observational birth cohort study (Boyd et al., 2013), have previously been used to contribute to the evidence regarding the ‘when’ and ‘what’ of complementary feeding practices. Regarding the former, late introduction of lumpy (chewy) foods, after 9 months of age, was related to higher levels of feeding difficulties and lower intakes of vegetables in mid-childhood (Coulthard, Harris, & Emmett, 2009; Northstone, Emmett, Nethersole, & ALSPAC Study Team, 2001). Two investigations of the ‘what’ aspect were made by comparing food and nutrient intakes of 3-year-old ‘picky’ children with those of ‘non-picky’ children. The diets of picky children provided lower intakes of fibre (Taylor, Northstone, Wernimont, & Emmett, 2016) and slightly lower intakes of protein, iron and zinc (Taylor, Northstone, Wernimont, & Emmett, 2016) than those of non-picky eaters. The children who were picky ate fewer vegetables and less meat than the non-picky children, contributing to the nutrient differences found. Furthermore, the lower fibre intake was associated with a greater likelihood of hard stools in picky than non-picky children (C. M. Taylor et al., 2016a,b). These findings add to the evidence that there are important consequences for the nutrition and well-being of children who are picky eaters. There is still a need, however, to understand ‘how’ factors relating to the child and the parents during complementary feeding may interact and nudge a child towards being a picky eater. Understanding these relationships could provide evidence for the development of preventive strategies that parents could employ with their young children.

The aim of this study is to investigate early life antecedents of parentally perceived picky eating behaviour in 3-year-old children in ALSPAC. The antecedents will be investigated in three sections: the first relating to the pregnancy including maternal diet; the second covering feeding behaviours and complementary feeding during the first year of life including breastfeeding; the third assessing feeding practices, behaviours and attitudes in the second year of life. A final model will combine the variables from each section that are associated with later picky eating to determine which is the most influential.

## 2 Subjects and methods

### 2.1 The ALSPAC cohort

ALSPAC is a longitudinal population-based prospective study investigating environmental and genetic influences on the health, behaviour and development of children. All pregnant women in the former Avon Health Authority with an expected delivery date between April 1991 and December 1992 were eligible for the study; 14,541 pregnant women were initially enrolled, resulting in a cohort of 14,062 live births with 13,988 alive at 1 year of age (Boyd et al., 2013). The social and demographic characteristics of this cohort were similar to those found in UK national census surveys (Fraser et al., 2013). Further details of ALSPAC are available at www.bris.ac.uk/alspac and the study website contains details of all the data that are available through a fully searchable data dictionary (http://www.bris.ac.uk/alspac/researchers/data-access/data-dictionary). Ethics approval for the study was obtained from the ALSPAC Ethics and Law Committee and the Local Research Ethics Committees. The primary data collection was through self-completion postal questionnaires.

### 2.2 Defining picky eating

The primary caregiver (usually the mother) received a questionnaire about her child at age 38 months. A single question similar to those used in several recent studies (C. M. Taylor et al., 2015) was asked: ‘Does your child have definite likes and dislikes as far as food is concerned?’ and possible answers were No/Yes, quite choosy/Yes, very choosy. This was used to define 3 parentally perceived categories of ‘picky eating’ status: *not picky* (45.2%), *somewhat picky* (40.1%) and *very picky* (14.7%) (C. M. Taylor et al., 2015). This measure has not been validated but is similar to those used in several recent studies (Goh & Jacob, 2012; Jani Mehta, Mallan, Mihrshahi, Mandalika, & Daniels, 2014; Mascola, Bryson, & Agras, 2010; Orun, Erdil, Cetinkaya, Tufan, & Yalcin, 2012) and shows strong associations with dietary intakes in the children (C.M. Taylor et al., 2016a,b).

### 2.3 Complementary feeding and child feeding behaviour

A series of questionnaires about the child throughout infancy were sent to the primary caregiver for completion. The full questionnaires are available from the study website (http://www.bristol.ac.uk/alspac/researchers/resources-available/data-details/questionnaires/). The wording of the questions and frequency of the answers used in this analysis are shown in Table 1.

**Table 1:** Questions asked to parents about feeding their child at different ages. Answers used in the regression models, grouped as shown for analysis, with frequency of occurrence in the whole cohort.

Questions as asked	Answer categories	Overall percent
**Child aged 4 weeks**		
Please indicate if your baby has had the following feeding behaviours:		
a) Weak sucking	Always/Sometimes	8.9
	Occasionally/No not at all	91.1 Reference
b) Choking	Always/Sometimes	26.5
	Occasionally/No not at all	73.5 Reference
c) Slow feeding	Always/Sometimes	24.7
	Occasionally/No not at all	75.3 Reference
d) Taking only small quantities at each fed	Always/Sometimes	29.1
	Occasionally/No not at all	70.9 Reference
Do you feel your baby is difficult to feed?	Yes, very/Yes, quite difficult	11.4
	No, not difficult	88.6 Reference
**Child aged 6 months**		
Is the baby fed 'on demand', i.e. whenever he/she is hungry?	Always/Sometimes	83.2
	No not at all	16.8 Reference
Please indicate if your baby had any of the following feeding behaviours and when they occurred:		
a) Slow feeding	Yes 0–3 months/Yes 4–6 months	28.9
	No not at all	71.1 Reference
b) Choking	Yes 0–3 months/Yes 4–6 months	22.3
	No not at all	77.7 Reference
c) Taking only small quantities at each fed	Yes 0–3 months/Yes 4–6 months	33.8
	No not at all	66.2 Reference
Do you feel you have ever had any difficulty feeding your baby?	Yes, great/Yes, some difficulties	35.4
	No, no difficulties	64.6 Reference
Has your baby refused to take solids before 6 months of age	Yes	21.6
	No	78.4 Reference
Breast feeding duration	Never	21.4
	<3 months	22.6
	3–5 months	17.3
	6 months or more	38.7 Reference
Age solid foods introduced	0–3 months	71.9
	4 months	25.0
	5 months or more	3.1 Reference
How often nowadays does your baby have?		
Prepared baby foods (from a jar, tin or packet) at 6 months, listing meat, fish, vegetable, fruit or milk pudding	Not answered	6.7
	22 times or more per week	11.5
	15-21 times per week	21.6
	8-14 times per week	32.1
	1-7 times per week	20.0
	None	8.1 Reference
Vegetables (cooked or raw) eaten at 6 months	Not answered	4.7
	8 times or more per week	12.2
	7 times per week	19.8
	1-6 times per week	44.9
	None	18.4 Reference
Fresh fruit eaten at 6 months	Not answered	1.6
	7 times or more per week	6.4
	1-6 times per week	36.0
	None	56.0 Reference
**Child aged 15 month**s		
Is he/she fed ‘on demand’, i.e. whenever he/she is hungry	Yes, always	14.4
	Yes, some of the time	53.2
	No, not at all	32.3 Reference
Babies first solid meals are usually a puree. When did your child first start having meals with lumps in?	Before 6 months	11.9
	Between 6 and 9 months	70.2 Reference
	10 months or more	17.9
Do you feel that you have had any difficulty feeding him/her in the past year?	Yes	40.6
	No	59.4 Reference
Since he/she was 6 months old has he/she at any time:		
a) Refused to eat the right foods?	Yes, worried me greatly	3.0
	Yes, worried me a bit	17.9
	Yes, but did not worry me	29.1
	No, did not happen	50.0 Reference
b) Been choosy with food?	Yes, worried me greatly	2.7
	Yes, worried me a bit	15.2
	Yes, but did not worry me	38.2
	No, did not happen	43.9 Reference
c) Not eaten enough food?	Yes, worried me greatly	6.4
	Yes, worried me a bit	24.4
	Yes, but did not worry me	25.6
	No, did not happen	43.6 Reference
How often nowadays does your child have?		
Number of times prepared baby/toddler or junior foods (from a jar, tin or packet) eaten	10 or more per week	8.1
	7-9 per week	4.6
	1-6 per week	17.0
	none	70.3 Reference
Number of times family meat/fish eaten	10 or more per week	9.8
	7-9 per week	37.3
	1-6 per week	43.6
	none	9.3 Reference
Number of times vegetables eaten	10 or more per week	17.9
	7-9 per week	45.9
	1-6 per week	29.7
	none	6.6 Reference
Number of times raw fruit eaten	10 or more per week	18.0
	7-9 per week	27.3
	1-6 per week	46.6
	none	8.1 Reference
For the main meal of the day does he/she eat:		
a) The same food as you?	No answer	2.2
	Always/almost always	67.0
	Sometimes	27.1
	Never or rarely	3.7 Reference
b) A ready-prepared meal out of a packet or tin?	No answer	13.4
	Always/almost always	2.6
	Sometimes	25.4
	Never or rarely	58.5 Reference

### 2.4 Maternal, pregnancy and background variables

Data from postal questionnaires in pregnancy and after the birth of the child were used to obtain maternal and demographic variables. These included parity (0, 1, ≥2), maternal age at delivery (≤20, 21–25, 26–30, >30 years of age) and highest educational attainment summarized as one of five categories (None; Vocational; Ordinary Level Certificate of School Education usually taken at 16 years of age; Advanced Level Certificate usually taken at 18 years of age; Degree). Pre-pregnancy body mass index was categorised as <20, 20–24.99, 25–29.99 and ≥ 30 kg/m^2^.

Dietary patterns at 32 weeks of pregnancy were derived from data collected by food frequency questionnaire (FFQ) covering foods eaten nowadays (Rogers & Emmett, 1998). There were 5 separate questions for meat, 3 for fish, 6 for vegetables, 1 for fresh fruit and 6 for sweet foods each with examples. Scores were obtained by principal components analysis (PCA) and five dietary patterns were identified: healthy (characterised by high intakes of salad, fruit, rice, pasta, fish, fruit juices, non-white bread), traditional (characterised by high intakes of meat, vegetables), processed (characterised by high intakes of meat pies, sausages, pizza, chips, crisps), confectionery (characterised by high intakes of biscuits, chocolate, sweets, cakes, puddings) and vegetarian (characterised by high intakes of pulses, nuts, herbal tea) (Northstone, Emmett, & Rogers, 2008). Each woman had a score for each pattern independently and the scores were grouped into quartiles for each pattern. In addition, the estimated weights of fruits and vegetables, meat and fish and sweet foods consumed by the mother in pregnancy were calculated from the FFQ and grouped into quartiles of intake (Rogers & Emmett, 1998).

Measures of maternal anxiety were obtained using the Crown–Crisp anxiety subscale (score 0–16) (Crown & Crisp, 1979) and of maternal depression using the Edinburgh Postnatal Depression Score (score 0–29) (Cox, Holden, & Sagovsky, 1987). Both scores were collected at 18 and 32 weeks of pregnancy, and at 8 weeks, and 8 and 21 months postpartum. High levels of symptoms were a score of ≥9 for anxiety and ≥13 for depression. The mother was also asked if she had ever had anorexia nervosa or bulimia (yes/no).

The sex and birth weight of the child (grouped as ≤2500, 2501–3000, 3001–3500, 3501–4000, >4000 g) were obtained from medical records.

### 2.5 Statistical analysis

Statistical analysis was carried out with SPSS v23 (IBM Corp.) on singletons only.

The complementary feeding and child behaviour variables listed in Table 1 were each tested in univariate analysis and found to be associated with ‘picky eating’ status (p < 0.001). They were therefore used in the multinomial logistic regression models, below. A flow diagram of participants and numbers available for each model is given in Supplemental figure 1.

A minimally adjusted regression model with the three levels of picky eating as the dependent variables included demographic and perinatal variables (age and education status of the mother, parity, sex of the child and birth weight (grouped)) as confounders, and was the basis for all the other models at three life stages. Maternal anxiety and depressive symptoms at each age measured were added separately to the minimally adjusted regression models to test if they should be included in the fully adjusted models: all (except depressive symptoms at 32 weeks gestation) were associated with at least one of the picky eating outcomes.

Three separate regression models (Models 1–3) were built to investigate the relative effects of influences from different stages of early life that were associated with picky eating in univariate analyses. Model 1 assessed maternal factors around pregnancy: pre-pregnancy BMI, symptoms of anxiety and depressive symptoms in pregnancy, and either dietary pattern scores in pregnancy (Model 1a) or intake of fruits, vegetables, meat/fish and sweet foods (Model 1b). Model 2 assessed factors occurring in the first year of life: weak sucking, choking, slow feeding, taking small quantities and difficulty to feed at 4 weeks, duration of breastfeeding, age of introduction to solid foods, choking, refusal of solid foods, intake of commercial baby foods, raw fruit and fresh vegetables, difficulty feeding at 6 months and age at introduction of lumpy foods, maternal symptoms of anxiety and depression at 8 weeks and 8 months postpartum. Model 3 assessed factors in the second year of life: difficulty in feeding, worries over child refusing food or being choosy with food or not eating enough at 15 months, maternal symptoms of anxiety and depression at 21 months postpartum, intakes of commercial baby foods or family foods (meat, fruit and vegetables) at 15 months (Model 3a), then including the child eating the same meal as mother and the child eating ready-prepared foods (Model 3b).

The models were then combined in a final model to determine independent influences on the likelihood of a child being a picky eater at age 3 years. The final model included the basic demographic and perinatal variables from the minimal model plus all variables that were significant at p ≤ 0.05 in the previous models.

## 3 Results

The minimally adjusted regression model accounted for 1.8% of the variation in whether the child was perceived as a picky eater at 38 months. Birthweight, parity and maternal education were associated with the child being a *very* picky eater; children who were first born or had a mother with a degree were positively associated; those with a high birthweight (≤4000g) were negatively associated (Table S1).

### 3.1 Pregnancy

The addition to the minimal model of maternal anxiety and depressive symptoms at two time points in pregnancy increased the variance explained slightly to 2.3%. Further adjustment for other variables in pregnancy (Table 2) increased the variance explained to 2.9% (Model 1a). The mother being overweight pre-pregnancy, her anxiety symptoms at 18 weeks, but not at 32 weeks of pregnancy, and high scores on a traditional or confectionery dietary pattern at 32 weeks were associated with picky eating status in Model 1a (Table 2). There were no associations with the other three dietary patterns. If mothers had many symptoms of anxiety the children were 47% more likely to be *very* picky eaters or if mothers had high scores on a ‘confectionery’ dietary pattern 29% more likely, while if mothers were overweight or had high scores on a ‘traditional’ dietary pattern *very* picky eating was less likely in the children. Underweight mothers were more likely to have a *somewhat* picky child and high scores on the ‘processed’ pattern were associated with a 21% increase in the likelihood of the child being *somewhat* picky. The mother ever having depressive symptoms, or anorexia nervosa or bulimia, was not related to her child being a picky eater. When maternal food group intake was assessed in model 1b instead of the dietary patterns the only food group associated with picky eating status was sweet foods, where the highest intake quartile was predictive of the child being a *very* (OR 1.35 (CI 1.09, 1.67), p = 0.006) or *somewhat* (OR 1.33 (CI 1.44, 1.56), p < 0.001) picky eater. There were no associations with meat/fish, fruit or vegetable intake quartiles.

**Table 2:** Model 1a: Antecedents during pregnancy of picky eating status at 3 years of age (minimally adjusted model^a^ plus pregnancy variables as shown (n = 6561)).

Predictor variable (reference category)	Predictor category	Child somewhat picky at 38 months			Child Very Picky at 38 months		
		OR	95% CI	P value	OR	95% CI	P value
**Pre-pregnancy BMI (20–24.99)**	<**20**	**1.16**	**1.02, 1.33**	**0.026**	1.08	0.91, 1.29	0.39
	**25–29.99**	0.94	0.80, 1.10	0.46	**0.75**	**0.59, 0.94**	**0.009**
	≥30	0.87	0.68, 1.13	0.30	0.79	0.55, 1.15	0.22
**Anxiety symptoms**							
**18 weeks (no)**	**Yes**	1.19	0.97, 1.46	0.095	**1.47**	**1.13, 1.91**	**0.005**
32 weeks (no)	Yes	1.07	0.88, 1.30	0.51	1.13	0.87, 1.47	0.36
Depressive symptoms							
18 weeks (no)	Yes	1.06	0.85, 1.32	0.63	1.00	0.74, 1.34	0.98
32 weeks (no)	Yes	0.94	0.77, 1.16	0.59	0.91	0.67, 1.20	0.50
Eating disorders							
Bulimia (no)	Yes	0.88	0.61, 1.28	0.52	1.05	0.64, 1.72	0.86
Anorexia Nervosa (no)	Yes	1.02	0.69, 1.52	0.92	0.79	0.45, 1.41	0.43
Dietary patterns							
Healthy (bottom quartile)	Top quartile	1.12	0.94, 1.35	0.20	0.85	0.66, 1.09	0.19
**Traditional (bottom quartile)**	**Top quartile**	0.90	0.77, 1.04	0.16	**0.80**	**0.54, 0.98**	**0.032**
**Processed (bottom quartile)**	**Top quartile**	**1.21**	**1.03, 1.42**	**0.018**	1.23	0.99, 1.53	0.063
**Confectionery (bottom quartile)**	**Top quartile**	**1.32**	**1.14, 1.54**	<**0.001**	**1.29**	**1.04, 1.59**	**0.018**
Vegetarian (bottom quartile)	Top quartile	1.05	0.90, 1.22	0.57	1.16	0.94, 1.43	0.19

Reference category: Not a picky eater at 38 months.

Model 1a explains 2.9% of the variance.

a Background demographic variables include: age and education status of the mother, parity, sex of the child and birth weight (grouped).

### 3.2 First year

The minimally adjusted model with the addition of variables from the first year of life (Model 2) explained 6.7% of the variance in picky eating status (Table 3). As shown in Table 1, it should be noted that almost all infants were introduced to solid foods by 4 months of age as recommended in the UK in the 1990s. The strongest predictor of the child being a *very* picky eater at 38 months was the late introduction of lumpy foods to the infant (81% more likely), followed by the child having refused solids before 6 months (63%), the child being fed on demand (44%), the mother indicating that she had found the infant difficult to feed by 6 months (33%) and the child showing signs of choking by 4 weeks was weakly associated. Both the child having weak sucking by 4 weeks and being introduced to lumpy food before 6 months were weakly protective. The mother having high levels of anxiety symptoms at 8 weeks postpartum was weakly associated with *very* picky eating being 43% more likely; there was no association with anxiety symptoms at 8 months postpartum. The child being a *very* picky eater was not associated with maternal depressive symptoms at 8 weeks or 8 months postpartum, breastfeeding duration, the age of introduction of solids or the feeding of baby foods, fresh vegetables or raw fruit at 6 months. There were, however, some associations with the child being *somewhat* picky (Table 3): not being breastfed and regularly eating vegetables at 6 months of age were weakly negatively associated.

**Table 3:** Model 2: Antecedents during first year of life of picky eating status at 3 years of age (minimally adjusted model^a^ plus first year variables as shown (n = 5758)).

Variable [Reference category]	Predictor category	Child *somewhat* picky at 38 months			Child *very* picky at 38 months		
		OR	95% CI	P value	OR	95% CI	P value
**Weak sucking by 4 weeks (no)**	**Yes**	0.95	0.76, 1.19	0.67	**0.70**	**0.52, 0.95**	**0.021**
**Choking by 4 weeks (no)**	**Yes**	1.08	0.95, 1.24	0.25	**1.24**	**1.03, 1.49**	**0.020**
Slow feeding by 4 weeks (no)	Yes	1.02	0.88, 1.19	0.79	1.21	0.98, 1.48	0.071
Small quantities by 4 weeks (no)	Yes	1.13	0.98, 1.30	0.09	1.04	0.86, 1.26	0.66
Difficult to feed at 4 weeks (no)	Yes	1.01	0.82, 1.23	0.96	1.11	0.85, 1.44	0.46
Age solids introduced (5 months or more)	0–3 months	1.10	0.78, 1.56	0.58	1.05	0.67, 1.66	0.83
	4 months	1.11	0.78, 1.58	0.56	1.01	0.64, 1.62	0.96
**Fed on demand at 6 months (no)**	**Yes**	**1.20**	**1.03, 1.41**	**0.020**	**1.44**	**1.15, 1.81**	**0.001**
**Difficulty to feed by 6 months (no)**	**Yes**	1.11	0.97, 1.27	0.15	**1.33**	**1.11, 1.60**	**0.002**
Slow feeding by 6 months (no)	Yes	1.14	0.98, 1.33	0.09	1.09	0.89, 1.34	0.41
Small quantities by 6 months (no)	Yes	1.09	0.95, 1.25	0.24	0.93	0.77, 1.13	0.48
Choking by 6 months (no)	Yes	1.04	0.90, 1.21	0.58	1.15	0.95, 1.40	0.15
**Refused solids by 6 months (no)**	**Yes**	**1.18**	**1.02, 1.38**	**0.030**	**1.63**	**1.34, 1.98**	<**0.001**
Duration of breast feeding (6 months +)	Never	**0.82**	**0.69, 0.98**	**0.025**	0.94	0.74, 1.19	0.62
	<3 months	0.90	0.76, 1.06	0.19	0.92	0.74, 1.15	0.45
	3–5 months	0.91	0.77, 1.07	0.25	0.86	0.68, 1.09	0.21
Baby food at 6 months (none)	Not Answered	1.15	0.85, 1.57	0.37	1.17	0.76, 1.80	0.47
	22x +/week	1.23	0.93, 1.61	0.15	1.89	0.96, 2.02	0.08
	15–21x/week	1.10	0.86, 1.41	0.46	1.12	0.79, 1.58	0.53
	8–14x/week	1.23	0.97, 1.56	0.08	1.19	0.86, 1.65	0.30
	1–7x/week	1.12	0.88, 1.43	0.34	0.99	0.71, 1.39	0.96
**Vegetables eaten at 6 months (none)**	Not answered	0.84	0.61, 1.14	0.25	0.71	0.46, 1.11	0.13
	8x +/week	0.81	0.64, 1.03	0.08	0.75	0.55, 1.04	0.09
	**7x/week**	**0.79**	**0.65, 0.97**	**0.021**	0.81	0.62, 1.05	0.11
	1–6x/week	0.91	0.78, 1.08	0.28	0.91	0.73, 1.13	0.40
Raw fruit eaten at 6 months (none)	Not answered	0.73	0.45, 1.19	0.21	0.74	0.38, 1.44	0.38
	7x +/week	1.02	0.79, 1.30	0.91	0.91	0.64, 1.29	0.59
	1–6x/week	1.12	0.99, 1.28	0.08	0.93	0.78, 1.11	0.43
**Age introduced to lumps (6–9 months)**	<**6 months**	**0.76**	**0.63, 0.92**	**0.004**	**0.74**	**0.56, 0.97**	**0.030**
	**10 months** +	**1.20**	**1.02, 1.41**	**0.027**	**1.81**	**1.48, 2.20**	<**0.001**
**Maternal anxiety symptoms**							
**8 weeks postpartum (no)**	**Yes**	0.91	0.69, 1.21	0.53	**1.43**	**1.01, 2.02**	**0.043**
8 months postpartum (no)	Yes	1.11	0.85, 1.44	0.46	1.18	0.84, 1.66	0.33
Maternal depressive symptoms							
8 weeks postpartum (no)	**Yes**	**1.34**	**1.02, 1.75**	**0.03**	1.01	0.71, 1.43	0.97
8 months postpartum (no)	Yes	0.90	0.67, 1.19	0.45	1.09	0.76, 1.56	0.65

Model 2 explains 6.7% of the variance.

Reference: Child not a picky eater at 38 months.

a Background demographic variables used for minimal adjustments include: age and education status of the mother, parity, sex of the child and birth weight (grouped).

### 3.3 Second year

In model 3a the variables from the second year of life were added to the minimal model and the child's diet was assessed without accounting for type of main meal fed by the mother. In this model, explaining 19.2% of the variance, the feeding of baby food 10 or more times per week was associated with increased odds of being *somewhat* picky (OR 1.44 (CI 1.16, 1.79), p = 0.001) or *very* picky (OR 1.65 (CI 1.25, 2.18), p < 0.001). In Model 3b (Table 4) the type of main meal fed by the mother was added and 20.1% of the variation in picky eating status was explained. In this model, the feeding of baby food was no longer independently associated but children whose mothers gave them mostly ready prepared meals at 15 months were twice as likely as those who did not to be *very* picky. Furthermore, the child and the mother mostly or sometimes eating the same meal at 15 months was protective against picky eating and if the child ate raw fruit at 15 months this was also associated with less likelihood of being a *very* picky eater at 38 months. By far the strongest predictor was the child being choosy about food at 15 months; if the mother was greatly worried about this choosiness the child was 6 times more likely to be a *very* picky eater at 38 months than a non-choosy child would be, whereas if the mother indicated that the child was choosy but this did not worry her the child was 3 times more likely to be a *very* picky eater later. If the mother was greatly worried about the child refusing foods this was also associated with *very* picky eating, being 3 times more likely at 38 months than if the child did not refuse food. Associations with *somewhat* picky eating in model 3b were largely similar but slightly weaker than those with *very* picky eating.

**Table 4:** Model 3b: Antecedents during second year of life of picky eating status at 3 years of age (minimally adjusted model^a^ plus second year variables as shown (n = 6608)).

	Reference category	Child somewhat picky at 38 months			Child very picky at 38 months		
		OR	95% CI	P value	OR	95% CI	P value
**Fed on demand at 15 months (no)**	**Yes, always**	**1.28**	**1.07, 1.52**	**0.007**	**1.37**	**1.07, 1.76**	**0.013**
	Yes, sometimes	**1.27**	**1.13, 1.44**	<**0.001**	1.20	1.00, 1.43	0.053
**Difficulty to feed at 15 months (no)**	**Yes**	**1.33**	**1.16, 1.53**	<**0.001**	**1.81**	**1.49, 2.20**	<**0.001**
**Refusing food at 15 months (no)**	**Yes, greatly worried**	**1.84**	**1.02, 3.34**	**0.045**	**2.93**	**1.54, 5.57**	**0.001**
	**Yes, a bit worried**	**1.48**	**1.18, 1.87**	**0.001**	**2.03**	**1.50, 2.74**	<**0.001**
	**Yes, not worried**	1.13	0.97, 1.31	0.12	**1.32**	**1.06, 1.65**	**0.015**
**Choosy with food at 15 months (no)**	**Yes, greatly worried**	**1.86**	**1.02, 3.38**	**0.043**	**6.03**	**3.21, 11.33**	<**0.001**
	**Yes, a bit worried**	**2.39**	**1.87, 3.06**	<**0.001**	**4.56**	**3.30, 6.31**	<**0.001**
	**Yes, not worried**	**2.08**	**1.80, 2.39**	<**0.001**	**3.14**	**2.50, 3.93**	<**0.001**
Not eating enough food at 15 months (no)	Yes, greatly worried	1.09	0.79, 1.51	0.59	1.45	0.99, 2.14	0.059
	Yes, a bit worried	0.99	0.83, 1.17	0.89	0.94	0.73, 1.19	0.59
	Yes, not worried	1.01	0.87, 1.17	0.89	0.96	0.77, 1.20	0.71
**Baby food at 15 months (none)**	≥**10 x/week**	**1.30**	**1.03, 1.64**	**0.025**	1.08	0.79, 1.47	0.64
	7–9 x/week	1.22	0.91, 1.61	0.18	1.09	0.75, 1.57	0.67
	1–6x/week	1.07	0.92, 1.25	0.40	0.83	0.66, 1.04	0.11
Family meat/fish eaten at 15 months (none)	≥10 x/week	0.83	0.60, 1.14	0.25	0.74	0.48, 1.15	0.18
	7–9 x/week	0.88	0.66, 1.16	0.36	0.80	0.55, 1.17	0.25
	1–6 x/week	0.86	0.66, 1.14	0.30	0.75	0.52, 1.08	0.13
**Raw fruit eaten at 15 months (none)**	≥**10 x/week**	1.10	0.84, 1.14	0.51	**0.59**	**0.41, 0.84**	**0.004**
	**7**–**9 x/week**	1.00	0.78, 1.29	0.98	**0.57**	**0.41, 0.79**	**0.001**
	**1**–**6 x/week**	1.04	0.81, 1.32	0.77	**0.69**	**0.51, 0.94**	**0.020**
Vegetables eaten at 15 months (none)	≥10 x/week	0.78	0.54, 1.12	0.18	1.02	0.62, 1.65	0.95
	7–9 x/week	0.98	0.70, 1.38	0.92	1.29	0.82, 2.04	0.27
	1–6x/week	0.92	0.66, 1.29	0.64	1.19	0.76, 1.85	0.45
**Main meal same as mother at 15 months (no)**	No answer	0.67	0.39, 1.16	0.15	0.47	0.25, 0.87	0.017
	**Mostly**	**0.63**	**0.43, 0.92**	**0.015**	**0.26**	**0.18, 0.40**	<**0.001**
	**Sometimes**	0.82	0.56, 1.19	0.29	**0.41**	**0.27, 0.61**	<**0.001**
Main meal ready prepared at 15 months (no)	No answer	0.91	0.76, 1.08	0.27	0.98	0.76, 1.27	0.88
	**Mostly**	1.23	0.79, 1.91	0.36	**2.26**	**1.36, 3.75**	**0.002**
	Sometimes	0.97	0.84, 1.12	0.68	1.17	0.94, 1.41	0.17
Anxiety symptoms at 21 months postpartum (no)	Yes	0.91	0.71, 1.15	0.42	1.09	0.79, 1.49	0.61
Depressive symptoms at 21 months postpartum (no)	Yes	1.15	0.91, 1.47	0.25	1.05	0.75, 1.46	0.78

Reference: Child not a picky eater at 38 months.

Model 3b explains 20.1% of the variance.

a Background demographic variables include: age and education status of the mother, parity, sex of the child and birth weight (grouped).

### 3.4 Combined analysis

The final combined regression model was tested using the same minimal adjustments and retaining breastfeeding duration and variables from each of the previous models that had been associated with the outcome at p ≤ 0.05. The variation in picky eating status explained by this model was 21.5% (Table 5). The associations with the background variables (Supplementary Table 2) were very similar to those in the minimal model except that being first born was no longer associated with being *very* picky whereas being a boy increased the likelihood by 33% (p = 0.001). Of the pregnancy variables, there were no independent associations with *very* picky eating. However, the mother being underweight in pregnancy was weakly independently associated with the child being *somewhat* picky as was being in the highest quartile of either the processed or confectionery maternal dietary pattern. In the first year, late introduction of lumps remained predictive of a child being *very* picky (43% more likely), but there was no strong evidence for other independent associations. In the second year, choosiness at 15 months was a very strong predictor of the child being a *very* picky eater at 38 months and this was enhanced by the mother indicating that this worried her (7 times more likely if the mother was worried about choosiness but only 3 times more likely if she was not worried). It is noteworthy that a choosy child at 15 months was twice as likely to be *somewhat* picky at 38 months whether the mother was worried or not. Likewise, refusal of foods at 15 months was strongly associated with picky eating status if the mother was worried about it (3 times more likely), and slightly less so if she was not worried. Other variables which were independently predictive were overall difficulty to feed at 15 months (80%) and always fed on demand at that age (44%). The mother mostly using ready-prepared food for the child's main meal was associated with being 2½ times more likely to be *very* picky at 38 months. The child eating raw fruit and the mother mostly or sometimes feeding the child the same main meal as herself were associated with less picky eating at the later age.

**Table 5:** Final Model: Antecedents during pregnancy and first and second year of life of picky eating status at 3 years of age (minimally adjusted model^a^ plus all pregnancy, year 1 and year 2 variables that were significant at ≤0.05 as shown (n = 5952)).

Variable [Reference category]	Predictor category	Child somewhat picky at 38 months			Child Very Picky at 38 months		
		OR	95% CI	P value	OR	95% CI	P value
**Pre-pregnancy BMI (20**–**24.99)**	>**20**	**1.18**	**1.02, 1.36**	**0.026**	1.04	0.85, 1.27	0.73
	25–25.99	1.02	0.86, 1.22	0.80	0.89	0.69, 1.16	0.39
	≤30	1.03	0.78, 1.37	0.83	0.97	0.64, 1.46	0.87
Anxiety symptoms at 18 weeks of pregnancy (no)	Yes	1.17	0.97, 1.42	0.10	1.24	0.96, 1.61	0.10
Traditional dietary pattern (bottom quartile)	Top quartile	0.98	0.83, 1.16	0.82	0.99	0.78, 1.25	0.91
**Processed dietary pattern (bottom quartile)**	**Top quartile**	**1.21**	**1.03, 1.42**	**0.029**	1.16	0.90, 1.49	0.25
**Confectionery dietary pattern (bottom quartile)**	**Top quartile**	**1.20**	**1.02, 1.44**	**0.031**	1.04	0.81, 1.32	0.78
**Weak sucking at 4 weeks (no)**	**Yes**	0.94	0.76, 1.17	0.59	**0.74**	**0.55, 1.00**	**0.048**
Choking at 4 weeks (no)	Yes	1.05	0.92, 1.20	0.50	1.11	0.92, 1.34	0.27
Fed on demand at 6 months (no)	Yes	1.08	0.91, 1.27	0.37	1.19	0.93, 1.52	0.18
Difficulty to feed at 6 months (no)	Yes	0.99	0.87, 1.13	0.91	0.96	0.79, 1.16	0.68
**Age introduced to lumps (6–9 months)**	<6 months	0.84	0.70, 1.00	0.054	0.91	0.69, 1.20	0.51
	**10 months** +	1.14	0.97, 1.34	0.12	**1.43**	**1.16, 1.78**	**0.001**
Refused solids by 6 months (no)	Yes	0.98	0.84, 1.14	0.79	1.22	1.00, 1.49	0.053
Duration of breast feeding (6 months +)	Never	0.88	0.74, 1.05	0.15	1.08	0.84, 1.38	0.56
	<3 months	0.95	0.80, 1.12	0.52	1.02	0.81, 1.29	0.85
	3–5 months	0.88	0.74, 1.05	0.15	0.87	0.68, 1.11	0.26
Vegetables eaten at 6 months (none)	Not answered	0.94	0.71, 1.26	0.70	0.79	0.50, 1.24	0.31
	8x +/week	1.04	0.83, 1.31	0.74	1.07	0.77, 1.50	0.69
	7x/week	0.95	0.78, 1.16	0.64	1.15	0.87, 1.52	0.33
	1–6x/week	1.02	0.86, 1.20	0.82	1.19	0.94, 1.49	0.15
Anxiety symptoms at 8 weeks postpartum (no)	Yes	0.85	0.64, 1.13	0.27	1.10	0.76, 1.59	0.63
Depressive symptoms at 8 weeks postpartum (no)	Yes	1.30	0.99, 1.70	0.06	1.03	0.71, 1.49	0.90
**Fed on demand at 15 months (no)**	**Yes, always**	**1.22**	**1.00, 1.48**	**0.047**	**1.44**	**1.10, 1.88**	**0.008**
	**Yes, sometimes**	**1.20**	**1.05, 1.38**	**0.008**	1.15	0.95, 1.40	0.16
**Difficulty to feed at 15 months (no)**	**Yes**	**1.34**	**1.16, 1.55**	<**0.001**	**1.80**	**1.46, 2.21**	<**0.001**
**Refusing food at 15 months (no)**	**Yes, greatly worried**	1.80	0.97, 3.33	0.061	**3.14**	**1.63, 6.07**	**0.001**
	**Yes, a bit worried**	**1.49**	**1.17, 1.89**	**0.001**	**2.09**	**1.54, 2.83**	<**0.001**
	**Yes, not worried**	1.15	0.99, 1.34	0.069	**1.30**	**1.04, 1.64**	**0.024**
**Choosy with food at 15 months (no)**	**Yes, greatly worried**	**2.25**	**1.16, 4.38**	**0.017**	**7.41**	**3.69, 14.88**	<**0.001**
	**Yes, a bit worried**	**2.32**	**1.79, 3.00**	<**0.001**	**4.40**	**3.14, 6.15**	<**0.001**
	**Yes, not worried**	**2.05**	**1.77, 2.38**	<**0.001**	**3.10**	**2.45, 3.92**	<**0.001**
Baby food at 15 months (none)	≥10 x/week	1.24	0.96, 1.59	0.10	1.04	0.74, 1.46	0.82
	7–9 x/week	1.13	0.84, 1.52	0.43	1.00	0.68, 1.49	0.99
	1–6x/week	1.11	0.94, 1.31	0.24	0.85	0.67, 1.08	0.18
**Raw fruit eaten at 15 months (none)**	≥**10 x/week**	0.84	0.64, 1.09	0.19	**0.46**	**0.32, 0.65**	<**0.001**
	**7**–**9 x/week**	0.82	0.64, 1.06	0.13	**0.48**	**0.35, 0.67**	<**0.001**
	**1**–**6 x/week**	0.85	0.67, 1.08	0.12	**0.55**	**0.41, 0.75**	<**0.001**
**Main meal same as mother at 15 months (no)**	No answer	0.65	0.37, 1.16	0.14	0.51	0.26, 1.00	0.048
	**Mostly**	**0.56**	**0.37, 0.83**	**0.004**	**0.28**	**0.18, 0.43**	<**0.001**
	**Sometimes**	0.73	0.49, 1.09	<0.12	**0.42**	**0.27, 0.66**	<**0.001**
Main meal ready prepared at 15 months (no)	No answer	0.92	0.77, 1.11	0.40	1.04	0.79, 1.37	0.81
	Mostly	1.26	0.79, 2.02	0.33	**2.48**	**1.45, 4.24**	**0.001**
	Sometimes	0.93	0.80, 1.09	0.38	1.10	0.88, 1.36	0.41

Reference: Child not a picky eater at 38 months.

Final model explains 21.5% of the variance.

a Background demographic variables include: age and education status of the mother, parity, sex of the child and birth weight (grouped).

There were some weak associations of maternal anxiety or depressive symptoms at some time points with picky eating status (Table 2, Table 3), but there were no independent associations in the final model.

### 3.5 Following the choosy child from 15 months to 3 years of age

**Fig. 1:**
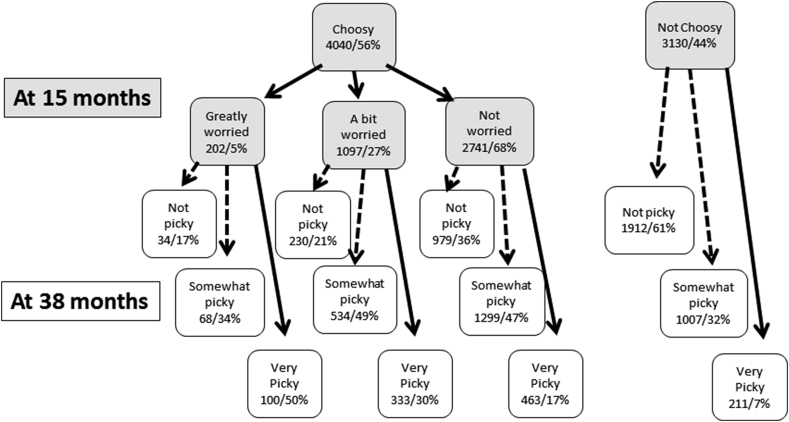
Relationships between early choosiness, maternal worry about choosiness and later picky eating behaviour.

Fig. 1 follows the children from age 15–38 months and shows that being thought to be choosy is very common at 15 months of age (56%) but that this does not always lead to later picky eating behaviour. Only 17% of choosy children at 15 months whose mothers were not worried about it were *very* picky at 38 months compared with 50% if their mothers were very worried, confirming that maternal worry about choosy behaviour in her child is a strong predictor of later picky eating.

## 4 Discussion

This study has investigated many of the recognised recommendations for complementary feeding in relation to parentally perceived picky eating. We found that in adjusted models, maternal and child factors in the second year of life, particularly maternal worry about feeding, were strongly associated with the child being perceived as a picky eater at 3 years of age with 21.5% of the variance explained by the model. Factors in pregnancy and over the first year of life were mostly not independently associated. The likelihood of a child being *very* picky at 3 years old was much higher if their mother was worried by their choosiness or refusal of foods in the second year of life. There was no evidence that longer duration of breastfeeding was independently associated with later picky eating. We have identified strategies and time points at which interventions are likely to be most effective in reducing the incidence of picky eating in children. These include: providing foods that help the child to learn to chew from 6 months or even before, and before 10 months of age; supporting mothers through the second year of life when children have a natural tendency to be wary of new foods; providing fresh fruit for the child, the mother often eating the same meal as the child and avoiding feeding ready-prepared baby foods.

A few other studies have investigated infant feeding as an antecedent to picky eating in young children, but most have only limited detail of how and what the infants were fed obtained retrospectively thus subject to recall bias. Children enrolled in the Generation R (n = 4779) study who were introduced to vegetables between 4 and 5 months had a lower food fussiness score at 4 years of age than those introduced after 6 months, and there were no meaningful associations with breastfeeding duration or the introduction of fruit or solids in general (de Barse et al., 2017). There findings are similar to those in to our present study. Shim, Kim, Mathai, and Strong Kids Research Team (2011) found that exclusive breast feeding for 6 months and introducing solids after 6 months were protective against food neophobia and lack of food variety in 2-3-year-old children. However, they had a small number of participants (n = 129) and were unable to assess separately children who always or sometimes showed these behaviours – an important differentiation in our study. We do not have comparable data from ALSPAC relating to introducing complementary foods at or after 6 months of age because almost all infants were introduced to solids at or before 4 months as recommended in the UK in the 1990s. There was no evidence that in this setting a very early age at introduction of solids was associated with occurrence of picky eating later. Furthermore, exclusive breast feeding to 6 months was not the recommended practice in the UK in the 1990s and we found no association of breastfeeding with being in the *very* picky group in our study. In the UK in the most up-to-date Infant Feeding Survey of 2010 only 23% of infants were exclusively breastfed at 6 weeks and 1% at 6 months of age; furthermore, solids had been introduced to 30% of infants by 4 months and 75% by 5 months of age (NHS Digital, 2012). This suggests that findings relating to the feeding practices prevalent in the 1990s are likely to continue to be relevant and the results from Generation R in the early 2000s support this (de Barse et al., 2017). The Feeding Infants and Toddlers Study (FITS) (van der Horst, Deming, Lesniauskas, Carr, & Reidy, 2016) is comparable with ALSPAC in assessing *somewhat* picky eaters separately from *very* picky eaters; information on breast feeding, but not complementary feeding was available and percentages of both *somewhat* and *very* picky eaters were higher compared with *not* picky when the child had never been breast fed. In contrast, in ALSPAC never-breast-fed children were less likely to be *somewhat* picky at 38 months and there was no association in *very* picky children (Table 3). It seems likely that once other feeding behaviours and practices are taken into consideration breast feeding is not a strong determinant of a child becoming a picky eater.

Some studies have investigated the texture of foods in relation to picky eating status but these have been cross-sectional studies not assessing specifically the timing of introduction of textures. The FITS study (van der Horst et al., 2016) found that both *somewhat* picky and *very* picky children were much more likely to resist or refuse certain food textures. In ALSPAC we have been able to take this a stage further back by showing that the age at which lumpy foods were introduced was independently associated with the child being either *somewhat* or *very* picky at a later age, suggesting that the introduction of lumps before 10 months of age is important (Le Reverend, Edelson, & Loret, 2014). In a randomised trial of modified baby-led weaning (BLISS trial), where infants are encouraged to feed themselves with pieces of food from 6 months onwards, the intervention group showed less food fussiness than the controls at 12 months (R. W. Taylor et al., 2017), confirming our findings.

Questions covering *refusal to eat food* and *not eating enough food* have been combined and used to define picky or fussy eating status in some studies (Cardona Cano et al., 2015; Machado, Dias, Lima, Campos, & Goncalves, 2016): in ALSPAC these were covered in the questionnaire completed when the child was 15 months old and incorporated in model 3 (Table 4). *Refusal to eat* but not *eating too little* was independently associated with the child being *somewhat* picky or *very* picky at 38 months by our definition based on the child having *definite likes and dislikes*; thus, it seems that different definitions identify groups of children with slightly different characteristics as picky eaters. The FITS study used a similar definition of picky eating to that in ALSPAC based on a single question to parents (van der Horst et al., 2016); furthermore, they asked if the child *resisted new foods* and found that this was much more likely in both *somewhat* and *very* picky than *not* picky children. The child *not eating enough* was not assessed in the FITS study. ALSPAC questioned a further aspect of these behaviours, namely whether the mother found the behaviour worrying. The odds of the child being *very* picky at a later age were strengthened if the mother was worried about the child *refusing food*.

The child being choosy with food at 15 months showed the strongest association with the child being defined as a picky eater approximately 2 years later based on having *definite likes and dislikes*. Even so this was not inevitable as about one-third of the children who were choosy at 15 months were not defined as picky at 38 months (Fig. 1). The extra dimension regarding the mothers' feelings of worry about their child's feeding behaviour is uniquely covered by ALSPAC and as such adds further insight into the development of picky eating behaviour. The mother being *greatly worried* about the child's choosiness at 15 months (5% of mothers with choosy children) was associated with greatly increased odds of the child being *very* picky but not of the child being *somewhat* picky at 38 months compared with the choosy child whose mother was not worried about it. It is possible that the mothers with a greater degree of worry had children with a greater propensity to choosiness for genetic or other reasons and that this accounts for their later picky eating behaviour. However, in a further analysis of this data we have shown that the mothers had greater odds of being either *greatly* or *a bit* worried if the child was first born, difficult to feed or refused solids by 6 months of age and there were no significant differences in associations with the antecedents related to the degree of maternal worry (P. M. Emmett, Hays, & Taylor, 2018). Both groups of worried mothers were more likely to introduce lumps late. These findings suggest that the inexperience of the mothers is an important factor.

Many studies have shown cross-sectionally that *pressure to eat* by a mother is associated with food fussiness and it could be that worried mothers are more likely to apply *pressure to eat*. Certainly, in a study by Gregory, Paxton, and Brozovic (2010) mothers concern about their child being underweight was associated with using *pressure to eat* when feeding their child. Child food fussiness was a predictor of the mother using *pressure to eat* (4% of the variance explained), but mothers' concern about underweight was a much stronger predictor (15% explained). The relationship between food fussiness or picky eating and *pressure to eat* is becoming clearer: in a study of 16-month-old twin pairs discordant for food fussiness (Harris, Fildes, Mallan, & Llewellyn, 2016) mothers used *pressure to eat* and *food rewards* more often with the fussier twin, suggesting that parents respond to their children by tailoring their feeding practices to the child's behaviour. Further evidence from Generation R found that preschool fussy eating was independently associated with parental use of *pressure to eat* in 4-year-olds and *pressure to eat* at 4 years was independently associated with more fussiness in the children when age 6 years (Jansen et al., 2017). The association between preschool fussy eating and parental *pressure to eat* was stronger than that between *pressure to eat* and later fussy eating.

Parental anxiety and depression in pregnancy and during the child's early life has been investigated in the Generation R study (de Barse et al., 2016): maternal anxiety and depression measured by BSI scores in pregnancy and 3 years later were associated with higher scores for the child on a fussy eating scale at 4 years. However, no account was taken of feeding behaviours and practices in infancy and toddlerhood and anxiety and depression were assessed in separate models. We used categorical variables rather than continuous scores for our analysis: although we found associations of both anxiety and depressive symptoms with the child being picky in the minimally adjusted model, very little of the variance was explained and these associations were mostly not robust to adjustment for other variables. Maternal anxiety symptoms in pregnancy and at 8 weeks postpartum were associated with picky eating in models 1 and 2, respectively, but were not independent of later feeding behaviours and practices in the final combined model. The presence of maternal depressive symptoms at 8 weeks postpartum was associated with the child being *somewhat* picky later, but there were no other associations with depressive symptoms. Our results suggest feeding practices and behaviours are much stronger determinants of picky eating behaviour in children than maternal anxiety or depression. There were no strong independent associations of maternal diet in pregnancy with picky eating at age 3 years although this may have been due to imprecision inherant in dietary assessment using an FFQ.

We found that the types of foods consumed at 15 months (Table 4) were associated with later picky eating status; the child eating fresh fruit and eating the same meal as the mother appeared to be protective against later picky eating. The child being fed ready-prepared main meals, especially baby foods, at 15 months tended to increase the likelihood of later picky eating. It has long been recognised that parents can act as role models to their children by eating healthy foods with them (Benton, 2004; Schwartz et al., 2011). These findings suggest that providing relatively simple instructions to parents about eating the types of foods they wish their pre-school child to eat at the same time as the child could help to mitigate the development of picky eating. The advice should emphasise using a variety of home-prepared fresh foods and eating these together as a family as often as possible.

We found that where the mothers indicated that the child was fed on demand at 15 months the likelihood of picky eating was increased. We have been unable to find any other study that has asked this question and as we did not define its meaning to the parents we are not sure how they interpreted it. It may suggest that the child is more likely to be given foods between meals when asking for something rather than the meals being planned or that the parents perhaps provide the foods they know the child likes thus limiting the introduction of new or unprefered foods. This aspect may be worth further exploration.

The strengths of this study include: (1) a single question about child choosiness which did not invite the parents to define picky eating for themselves was used to define picky eating status; (2) a non-picky comparison group was included; (3) parental questionnaires were completed prospectively therefore not subject to recall bias; (4) no other studies have comparable data to this in such large numbers of children. Limitations include: (1) the question to assess picky eating status did not cover the full range of ‘picky eating’ traits and was based on parental perception not professional judgement; (2) some of the picky eating groups contained relatively small numbers of children; (3) there was some attrition and incomplete data collection; (4) data were collected by postal questionnaires from untrained parents and as such may be biased by their understanding of the various questions and their subjectivity in answering; (5) the study was carried out in one geographically defined area of the UK in 1990s, which may limit generalisability, although comparisons with children's dietary intakes collected throughout the UK in 1990s and in 2008–11 have shown very similar nutrient and food group intakes to those in these children (Emmett, Rogers, Symes, & ALSPAC Study Team, 2002; Emmett & Jones, 2015); (6) since some of the children may have been considered to be picky eaters very early in life, there is the possibility of reverse causation (e.g. parents of a child whom they perceive to be picky may delay giving lumpy foods); (7) a small proportion of the children who were identified as picky eaters may have had, or have gone on to have, severe feeding difficulties, which we were not able to identify and which could be a cause of maternal worry; (8) there may be unmeasured confounding that we were unable to account for.

In conclusion, this study has identified modifiable feeding practices associated with later ‘picky eating’ behaviour in young children and thus provides evidence on which to base advice and support to parents designed to limit the development of this behaviour. Key points for parents are to introduce lumpy foods to infants at 6 months and not later than 9 months of age, to use fresh foods particularly fruit during the complementary feeding process, and to eat the same meal with the child whenever possible. When children show signs of being choosy this is a natural phase in their development and parents should persist in offering but not forcing a variety of fresh foods. Health professionals need to support parents with consistent advice especially during the choosy phase of complementary feeding. Their support should be designed to increase confidence and decrease worry in the parents around their ability to feed their children adequately.

## Conflicts of interests

PME has from time to time received research funding and consultancy funding from Nestlé Nutrition, Pfizer Nutrition Ltd, and Danone Baby Nutrition (Nutricia Ltd). NPH is an employee of Nestlé Nutrition (which provided funding for the research). CMT had no conflicts of interest related to the study.

## Sources of support

The UK Medical Research Council and the Wellcome Trust (Grant ref: 102215/2/13/2) and the University of Bristol provide core support for ALSPAC. A comprehensive list of grants funding ALSPAC is available on the ALSPAC website. This research was specifically funded by Nestlé Nutrition. CMT was supported by a Wellcome Trust Career Re-entry Fellowship (Grant ref: 104077/Z/14/Z).

## Authors' contributions

CMT, PME and NPH designed the research; PME analysed the data; PME wrote the paper with critical revisions from CMT and NPH; CMT and PME have primary responsibility for the final content and all authors; read and approved the final manuscript.

## Appendix A Supplementary data

Supplemental table 2 Supplemental table 2

Data Profile Data Profile
