# Supplementary material for: Antecedents of picky eating behaviour in young children
Source: Appetite. 2018 Nov 1;130:163–73. doi: 10.1016/j.appet.2018.07.032 (PMC6173797; doi:10.1016/j.appet.2018.07.032)
Supplement: Supplemental Figure 1 [file mmc1.pptx]

## Slide 1
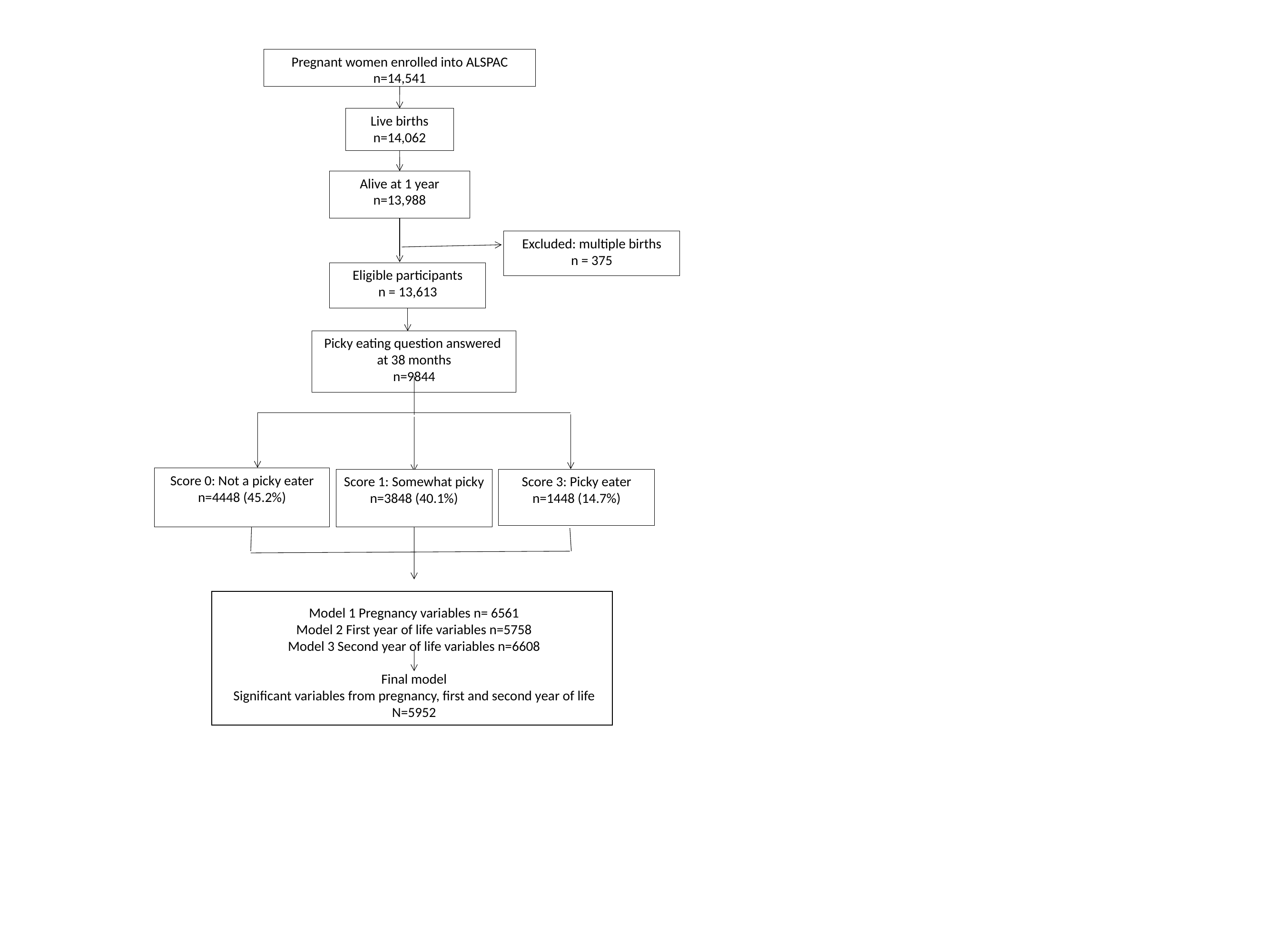

Pregnant women enrolled into ALSPAC
n=14,541
Live births
n=14,062
Alive at 1 year
n=13,988
Excluded: multiple births
n = 375
Eligible participants
n = 13,613
Picky eating question answered
at 38 months
n=9844
Score 0: Not a picky eater
n=4448 (45.2%)
Score 3: Picky eater
n=1448 (14.7%)
Score 1: Somewhat picky
n=3848 (40.1%)
Model 1 Pregnancy variables n= 6561
Model 2 First year of life variables n=5758
Model 3 Second year of life variables n=6608
Final model
Significant variables from pregnancy, first and second year of life
N=5952
