# Supplementary material for: Antecedents of picky eating behaviour in young children
Source: Appetite. 2018 Nov 1;130:163–73. doi: 10.1016/j.appet.2018.07.032 (PMC6173797; doi:10.1016/j.appet.2018.07.032)
Supplement: Supplemental table 1 [file mmc2.docx]

Supplemental Table 1. Minimal model: Antecedents during pregnancy of picky eating status at 3 years of age (n=7147).

| **Predictor variable (reference category)** | **Predictor**  **category** | **Child somewhat picky at 38 months** | | | **Child Very Picky at 38 months** | | |
| --- | --- | --- | --- | --- | --- | --- | --- |
|  |  | OR | 95% CI | P value | OR | 95% CI | P value |
|  |  |  |  |  |  |  |  |
| **Parity (2 or more)** | **0** | **1.35** | **1.16, 1.56** | **<0.001** | **1.35** | **1.10, 1.66** | **0.005** |
|  | **1** | **1.17** | **1.01, 1.36** | **0.035** | 1.11 | 0.91, 1.37 | 0.31 |
| **Maternal age at delivery (>30 years)** | **≤20** | **0.62** | **0.44, 0.87** | **0.005** | 0.65 | 0.41. 1.04 | 0.071 |
|  | **21-25** | **0.77** | **0.66, 0.91** | **0.002** | 0.81 | 0.65, 1.01 | 0.056 |
|  | 26-30 | 0.92 | 0.82, 1.03 | 0.16 | 0.92 | 0.78, 1.08 | 0.30 |
| **Birthweight (3001-3500 g)** | 2500g or less | 1.18 | 0.92, 1.52 | 0.19 | 1.36 | 0.99, 1.86 | 0.056 |
|  | **2501-3000g** | 1.11 | 0.94, 1.30 | 0.23 | **1.26** | **1.02, 1.55** | **0.033** |
|  | 3501-4000g | 1.03 | 0.91, 1.17 | 0.63 | 0.91 | 0.77, 1.07 | 0.25 |
|  | **4001g or more** | 0.92 | 0.78, 1.08 | 0.31 | **0.66** | **0.51, 0.84** | **0.001** |
| **Maternal Education (Low)** | **Degree** | **1.45** | **1.19, 1.76** | **<0.001** | **1.57** | **1.20, 2.05** | **0.001** |
|  | A level | 1.16 | 0.98, 1.38 | 0.089 | 1.21 | 0.95, 1.54 | 0.13 |
|  | O level | 0.95 | 0.91, 1.26 | 0.39 | 1.21 | 0.96, 1.51 | 0.11 |
|  | Vocational | 0.95 | 0.76, 1.19 | 0.67 | 1.20 | 0.89, 1.62 | 0.24 |
| Sex of child (Female) | Male | 0.95 | 0.86, 1.05 | 0.30 | 1.13 | 0.98, 1.30 | 0.091 |

Reference category: Not a picky eater at 38 months

Minimal model explains 1.8% of the variance
