# Supplementary material for: Antecedents of picky eating behaviour in young children
Source: Appetite. 2018 Nov 1;130:163–73. doi: 10.1016/j.appet.2018.07.032 (PMC6173797; doi:10.1016/j.appet.2018.07.032)
Supplement: Supplemental table 2 [file mmc3.docx]

Supplemental Table 2. Background antecedents of picky eating status at 3 years of age adjusted for all variables listed in table 5: (n=5952).

| **Predictor variable (reference category)** | **Predictor**  **category** | **Child somewhat picky at 38 months** | | | **Child Very Picky at 38 months** | | |
| --- | --- | --- | --- | --- | --- | --- | --- |
|  |  | OR | 95% CI | P value | OR | 95% CI | P value |
|  |  |  |  |  |  |  |  |
| **Parity (2 or more)** | **0** | **1.26** | **1.05, 1.51** | **0.014** | 1.00 | 0.77, 1.29 | 0.97 |
|  | **1** | **1.19** | **1.00, 1.42** | **0.046** | 1.09 | 0.85, 1.40 | 0.49 |
| **Maternal age at delivery (>30 years)** | **≤20** | **0.57** | **0.37, 0.86** | **0.008** | 0.61 | 0.33. 1.14 | 0.12 |
|  | **21-25** | **0.76** | **0.62, 0.91** | **0.004** | 0.83 | 0.63, 1.08 | 0.17 |
|  | 26-30 | 0.92 | 0.80, 1.05 | 0.21 | 0.94 | 0.77, 1.15 | 0.55 |
| **Birthweight (3001-3500 g)** | 2500g or less | 1.02 | 0.75, 1.39 | 0.89 | 1.24 | 0.83, 1.85 | 0.29 |
|  | 2501-3000g | 1.01 | 0.84, 1.22 | 0.93 | 1.11 | 0.86, 1.43 | 0.43 |
|  | 3501-4000g | 0.98 | 0.85, 1.13 | 0.82 | 0.85 | 0.70, 1.04 | 0.12 |
|  | **4001g or more** | 0.97 | 0.80, 1.17 | 0.74 | **0.66** | **0.49, 0.88** | **0.006** |
| **Maternal Education (Low)** | **Degree** | **1.35** | **1.06, 1.72** | **0.017** | **1.68** | **1.18, 2.39** | **0.004** |
|  | A level | 1.10 | 0.89, 1.36 | 0.39 | 1.26 | 0.92, 1.73 | 0.14 |
|  | **O level** | 1.11 | 0.92, 1.36 | 0.28 | **1.40** | **1.05, 1.87** | **0.023** |
|  | Vocational | 0.95 | 0.73, 1.24 | 0.71 | 1.23 | 0.85, 1.79 | 0.28 |
| Sex of child (Female) | **Male** | 1.04 | 0.92, 1.17 | 0.55 | **1.33** | **1.12, 1.58** | **0.001** |

Reference category: Not a picky eater at 38 months

Whole model explains 21.5% of the variance
